# Fed, not fasted: is carbohydrate mouth rinsing still ergogenic? A three-level meta-analysis

**DOI:** 10.1080/15502783.2025.2579027

**Published:** 2025-11-06

**Authors:** Hengzhi Deng, Xiaohan Fan, Ping Liu, Tianyu Song, Abdullah Al-Hadi Ahmad Fuaad, Nasnoor Juzaily Bin Mohd Nasiruddin, Mohamed Nashrudin Bin Naharudin

**Affiliations:** a Faculty of Sports and Exercise Science, University Malaya, Kuala Lumpur, Malaysia; b Faculty of Science, University Malaya, Kuala Lumpur, Malaysia

**Keywords:** Carbohydrate mouth rinsing, cognitive performance, exercise performance, fed state, meta-analysis

## Abstract

**Background:**

Carbohydrate (CHO) mouth rinsing improves performance by stimulating oral receptors linked to brain regions involved in motor control and motivation, without requiring digestive processing. Most research has focused on fasting states, but the effects of postprandial CHO mouth rinsing remain inconclusive. This study aims to synthesize existing studies on the impact of postprandial CHO mouth rinsing on exercise and cognitive performance, offering insights for future research and practical recommendations for athletes and coaches.

**Methods:**

Six databases (Pubmed, Web of Science, Cochrane Library, Embase, SciELO and SPORTDiscus) were searched up to March 2025 for randomized, placebo-controlled trials in healthy adults who performed exercise or cognitive tasks under fed conditions following CHO mouth rinsing. A three-level random-effects meta-analysis was performed for exercise performance, while narrative synthesis was applied for cognitive outcomes. For exercise performance, moderator and meta-regression analyses examined sex, training status, exercise modality, rinse composition and concentration, rinse duration, timing of food intake and pre-exercise dietary content.

**Results:**

Thirty-five articles met inclusion criteria: two assessed cognitive performance and thirty-three evaluated exercise performance. Overall, CHO mouth rinsing improved cognitive function under fed conditions compared with placebo, though certainty was very low. Meta-analysis showed a small but significant ergogenic effect on exercise performance (Hedges’ g = 0.18, 95% CI [0.09, 0.28], *p* < 0.01). Moderator and meta-regression analyses indicated that CHO mouth rinsing was more effective under fed conditions during aerobic exercise, when using maltodextrin solutions, rinsing for ≤10 s, and following a high-CHO meal.

**Conclusions:**

This systematic review and three-level meta-analysis provides evidence that CHO mouth rinsing is ergogenic under fed conditions, improving both exercise and cognitive performance, though the overall certainty of evidence is low. Practical applications include its potential use as a simple, noninvasive ergogenic aid, particularly when combined with specific exercise modalities and nutritional strategies.

## KEYPOINTS

- This study provides the first comprehensive summary of carbohydrate (CHO) mouth-rinsing effects on exercise and cognitive performance under fed conditions.
- A three-level meta-analysis with multiple validation methods was conducted to resolve the fasting vs. feeding debate, confirming the effectiveness of CHO mouth rinsing on exercise performance in a fed state.
- Although limited by sample size, available evidence indicates that CHO rinsing may also enhance cognitive performance under fed conditions, supporting a centrally mediated mechanism of action.
- Moderator analysis suggests that CHO rinsing is more effective under fed conditions when:
  - Applied during aerobic exercise
  - Using maltodextrin-based solutions
  - Rinsing for ≤10 seconds
  - Following a high-CHO pre-exercise meal

## 1. Introduction

Nutritional status prior to exercise is a decisive factor in determining athletic performance [1,2] . Among the macronutrients, carbohydrates (CHO) are widely regarded as the most effective substrate for supporting high-intensity exercise, owing to their capacity to sustain blood glucose, delay the onset of fatigue, and preserve glycogen stores in both muscle and liver [3–5]. These properties are well established in prolonged endurance events.

Interestingly, CHO ingestion has also been shown to enhance performance in exercise lasting less than 60 minutes, when metabolic limitations, such as hypoglycemia and glycogen depletion, are less likely to be the primary constraining factors [6–8]. This observation has led to the proposition that CHO may exert central, non-metabolic effects by stimulating oropharyngeal receptors that activate brain areas linked to reward, motivation, and motor control [6,9].

Building on these insights, researchers began to explore strategies to experimentally isolate such non-metabolic effects from gastrointestinal metabolism. Evidence suggests that the vast majority of pre-exercise nutritional interventions take place in acute situations [4]. In this context, Carter and colleagues introduced the concept of CHO mouth rinsing in 2004. Their pioneering study showed that simply rinsing the mouth with a CHO solution, without swallowing, could improve cycling performance [9]. Subsequent research has repeatedly confirmed its ergogenic potential under fasted conditions, such as overnight fasting of 8–12 hours, which minimize metabolic contributions and thereby allow central mechanisms to be more directly examined [10–13].

However, real-world competition rarely occurs in the fasted state. Athletes typically consume pre-event meals to maximize energy availability, which raises the question of whether CHO mouth rinse retains its performance-enhancing effects under postprandial (fed) conditions. While numerous reviews have summarized findings from fasted-state protocols, evidence in the fed state remains limited, fragmented, and inconclusive.

Beyond physical output, many sports, particularly those requiring rapid decision-making, reaction speed, or multi-tasking, also depend on cognitive function. CHO mouth rinse has been proposed to influence cognition through similar central neural mechanisms [12,14], yet no systematic review has specifically addressed its effects under fed conditions.

Therefore, this systematic review and meta-analysis aims to synthesize the available evidence on the effects of CHO mouth rinsing on both exercise and cognitive performance in postprandial states, defined as within 4 hours after food intake. By focusing exclusively on competition-relevant feeding conditions, we seek to bridge an important gap in the literature and provide actionable insights for athletes, coaches, and sport scientists.

## 2. Methods

This study was conducted in reference with the PRISMA (Preferred Reporting Items for Systematic Reviews and Meta-Analyses) guidelines [15]. The completed PRISMA 2020 checklist is available in **Electronic Supplementary Material Appendix S1**. This methodological framework builds upon our previously posted preprint [16], in which an earlier version of the analysis protocol was introduced. The current manuscript reflects updates and refinements following further validation and review.

### 2.1. Eligibility criteria

Studies were considered eligible if they met all of the following criteria: (1) randomized controlled trials published in peer-reviewed journals; (2) investigated the effects of CHO mouth rinsing as the intervention; (3) enrolled healthy adult human participants (≥18 years) who had consumed food within 4 hours prior to testing; (4) included a non-CHO control condition, such as a water mouth rinse or a non-caloric, taste-matched placebo rinse; (5) reported original experimental data; and (6) were published in English.

Studies were excluded if they met any of the following conditions: (1) the rinse solution did not contain CHO; (2) other active substances were included (e.g. caffeine, menthol) or the intervention involved ingestion rather than mouth rinsing; (3) participants fasted for more than four hours before testing; (4) feeding status was unclear (e.g. described only as “ate normally”); (5) the report was non-original (e.g. review articles, conference abstracts); or (6) essential methodological details were not provided.

The four-hour threshold for defining the postprandial (fed) state was selected based on previous experimental protocols [6,9] and is commonly adopted in exercise nutrition research. Although the composition of the preceding meal can influence gastric emptying and metabolic responses, this ≤4-hour window was considered to offer a practical and physiologically relevant balance, ensuring comparability across studies.

In line with the PICOs framework, our study design can be summarized as follows: Participants: healthy adults tested in the fed state (≤4 h after a meal); Intervention: CHO mouth rinse; Comparison: non-CHO controls (water rinse or non-caloric, taste-matched placebo); Outcomes: exercise and cognitive performance measures.

### 2.2. Data sources and search

A systematic search was performed in March 2025 across PubMed, Web of Science, Cochrane Library, Embase, SciELO, and SPORTDiscus. Two parallel strategies were used to identify studies related to either exercise or cognitive performance:

1. “carbohydrate mouth rinse” OR “carbohydrate oral rinse” OR “carbohydrate mouthwash” AND “exercise.”
2. “carbohydrate mouth rinse” OR “carbohydrate oral rinse” OR “carbohydrate mouthwash” AND “cognition.”

No date or filter restrictions were applied.

### 2.3. Data extraction

All retrieved records were exported to Excel and EndNote (v21) for de-duplication. Two independent reviewers screened titles, abstracts, and full texts. Any discrepancies were resolved through discussion and consensus. Extracted data included: sample size, sex, training status, exercise protocol, feeding time, rinse solution and duration, and key outcomes (e.g. time to exhaustion, mean power output). When data were not reported numerically, authors were contacted or data were extracted using WebPlotDigitizer (version 4.8; https://automeris.io/WebPlotDigitizer/).

### 2.4. Risk of bias assessment

Risk of bias was assessed using the Cochrane Risk of Bias 2 (RoB 2) tool [17], which evaluates potential bias across five domains: randomization process, deviations from intended interventions, missing outcome data, measurement of the outcome, and selection of the reported result. Two reviewers (D.H.Z. and F.X.H.) conducted the assessments independently. Any disagreements were resolved by discussion, with arbitration by a senior reviewer (M.N.N.) when necessary.

### 2.5. Statistical analysis

#### 2.5.1. Data extraction, synthesis and effect measures

Only performance-related outcomes were included in the meta-analysis. The primary effect size was Hedges’g (standardized mean difference), computed as the mean difference between the CHO and placebo conditions divided by the pooled standard deviation, with small-sample bias correction [18]. For crossover trials, the pooled SD was calculated as

$$SD_{\mathrm{pooled}}=\sqrt{\frac{S{D^{2}}_{\mathrm{CHO}}+S{D^{2}}_{\mathrm{PLA}}}{2}}$$

Where SD_CHO_ is the standard deviation from CHO mouth rinse group, and SD_PLA_ is the standard deviation from placebo group.

For parallel-group trials, pooled SD was computed as [18]:

$$SD_{\mathrm{pooled}}=\sqrt{\frac{\left(n_{\mathrm{CHO}}-1\right)\times SD_{CHO}^{2}+\left(n_{\mathrm{PLA}}-1\right)\times SD_{PLA}^{2}}{n_{\mathrm{CHO}}+n_{\mathrm{PLA}}-2}}$$

Where n_CHO_ is the reported sample size of the CHO mouth rinse group, and n_PLA_ is the reported sample size of the placebo group.

For the crossover trials, Hedges’ g was computed as [18]:

$$\mathrm{Hedg}e^{\prime }sg=\;\frac{\left(\;[M_{\mathrm{CHO}}\right]-\;[M_{\mathrm{PLA}}])}{SD_{\mathrm{pooled}}}\times (1-\frac{3}{4\left(N-1\right)-1}$$

Where M_CHO_ and M_PLA_ are the mean values of the CHO and placebo conditions, respectively, and N is the total sample size.

For parallel-group designs, Hedges’ g was computed as [18]:

$$\mathrm{Hedg}e^{\prime }sg=\;\frac{\left(\;[M_{\mathrm{CHO}}\right]-\;[M_{\mathrm{PLA}}])}{SD_{\mathrm{pooled}}}\times (1-\frac{3}{4\left(n_{\mathrm{CHO}}+n_{\mathrm{PLA}}-2\right)-1}$$

Where *g* was classified as *trivial* (0.2), *small* (0.2–0.5), *medium* (0.5–0.8), and *large* ( > 0.8) [19].

Standard errors (SE) for crossover studies were estimated with [18]:

$$\mathrm{SE}=\sqrt{\frac{1}{N}+\frac{g^{2}}{2N}}\times \sqrt{2\left(1-r\right)}$$

Where r is the correlation coefficient between the CHO and placebo conditions, which was rarely reported in the included studies. Following the Cochrane Handbook [20], we initially assumed r = 0.50. When available, more precise values were obtained either by directly contacting study authors or by estimating them from published figures, which indicated that most correlations ranged between 0.8 and 0.9. Accordingly, consistent with Oliveira-Silva et al. (2024) [21], who adopted r = 0.85 in a meta-analysis of crossover trials on CHO mouth rinse and exercise performance, we conducted the main analyses with r = 0.85 and reported those results. Sensitivity analyses were also performed with r = 0.20 (extreme) and r = 0.50 (conservative). For each analysis, a single *r* value was applied uniformly across all crossover outcomes (i.e. all outcomes at r = 0.85 for the main analyses, all at r = 0.50, and all at r = 0.20 for sensitivity checks), ensuring clarity and comparability.

For parallel-group designs, SE was computed as [22,23]:

$$\mathrm{SE}=\sqrt{\frac{n_{\mathrm{CHO}}+n_{\mathrm{PLA}}}{n_{\mathrm{CHO}}\times n_{\mathrm{PLA}}}+\frac{g^{2}}{2\left(n_{\mathrm{CHO}}+n_{\mathrm{PLA}}\right)}}$$

#### 2.5.2. Meta-analysis and heterogeneity

All quantitative syntheses were conducted using random-effects models to account for between-study variability [23,24]. Because several studies reported multiple outcomes, we employed a three-level meta-analytic framework [25–27] that partitioned variance into sampling error (level 1), within-study variance (level 2), and between-study variance (level 3) [27]. All coefficient tests and confidence intervals (CI) were computed using a t-distribution [28]. Parameter estimates were obtained via restricted maximum likelihood (REML), and prediction intervals were calculated to evaluate the expected range of effects in new studies [29,30].

Heterogeneity was quantified using the I^2^ statistic [31]: 0–25%: low; 25–50%: moderate; 50–75%: substantial; > 75%: considerable.

Additionally, the statistical power of the primary pooled effect was estimated to assess the likelihood of Type II errors. All power calculations were conducted using the metameta package [32] and applied consistently across subgroup and moderator analyses.

#### 2.5.3. Moderators and subgroup analysis

To explore potential sources of heterogeneity, moderator analyses were performed using both conventional three-level subgroup analyses (for categorical moderators) and meta-regression models (for both continuous and categorical moderators, with the latter coded as dummy variables). As meta-regression utilizes the full sample, it allows for a more comprehensive assessment of moderator effects and is generally considered more robust and informative [33–35]. It is generally recommended that meta-regression be conducted only when at least 10 studies are available, and that each subgroup in subgroup analyses includes at least 5 studies [36,37]. Moderators included: 1) participant sex; 2) training status; 3) exercise modality (aerobic vs anaerobic); 4) rinse solution type; 5) rinse duration; 6) meal composition; 7) CHO concentration; and 8) pre-exercise feeding time.

Based on previous participant categorization frameworks, participants were classified as untrained and trained (recreationally active, trained/developmental, well-trained/national level, elite/international level, and world-class) [38]. According to Mitchell et al. (2005) recommendations, the exercise types were classified into two broad categories: aerobic and anaerobic (strength or explosive) [39]. This simplified categorization was adopted to ensure sufficient sample sizes within each category, thereby maintaining adequate statistical power, and to reduce potential bias arising from overly granular or subjective classifications. Furthermore, synthesizing previous research classifications [40–42], high, medium, and low CHO meals were divided according to the proportion of CHO in total calories (high ≥60%, medium 31–59%, and low ≤30%). If the intake was not explicitly specified, it was classified as unspecified (NS). Since weight-normalized intake was not universally reported in the original studies, individual correction was not performed in this study.

CHO concentration and feeding time were treated as continuous variables in regression analyses. Mixed-effects meta-regression with REML was used for all moderator models [43], and best-fitting functional forms were selected using AICc [44]. All regression models were performed using the metafor package and later visualized with the ggplot2 package [45].

#### 2.5.4. Risk of publication bias and sensitivity analysis

The contour-enhanced funnel plot [46], along with Egger’s asymmetry test [47,48], was employed to assess publication bias (tests were only conducted when k ≥ 10) [43], with *p* > 0.05 indicating no significant publication bias. Funnel plots and Egger’s regression tests are primarily used to determine the symmetry of the overall effect size, either through subjective or quantitative measures, thereby assessing the risk of publication bias in the included studies. Given the complexity of three-level modeling, we did not apply traditional bias correction methods (e.g. Trim-and-Fill), but instead used sensitivity analyses to assess robustness.

Our sensitivity analysis was conducted on three-levels model. First, we conducted sensitivity analyses using different correlation coefficients to calculate the standard error. Second, we conducted a leave-one-out analysis, sequentially removing each study to assess whether any single study significantly influenced the overall pooled effect. Finally, we identified potential outliers in the outcome measures using the three-level meta-analysis and examined their influence on the overall effect size in the primary model. Specifically, in the three-level meta-analysis, Cook’s distance [49] and studentized residuals [50] were employed to diagnose leverage, outliers, and influential cases at the within-study level (level 2) and between-study level (level 3), respectively. Cases were flagged if their Hat and Cook’s distance values exceeded three times their respective means, or if their studentized residuals had absolute values greater than 3. The three-level meta-analysis was then repeated after excluding these outliers to assess the model’s stability.

### 2.6. Certainty of the evidence

The certainty of evidence was assessed using the Grading of Recommendations Assessment, Development, and Evaluation (GRADE) framework, which classifies evidence as “high,” “moderate,” “low,” or “very low” [51]. For outcomes synthesized quantitatively (exercise performance), we applied the standard GRADE domains: risk of bias, inconsistency, indirectness, imprecision, and publication bias. For cognitive outcomes, where meta-analysis was not feasible, we adopted the narrative-adapted GRADE approach proposed by Murad et al. (2017) [52], which considers similar domains but places greater emphasis on study design, sample size, consistency of findings, and methodological rigor. All GRADE assessments were performed independently by one reviewer and verified by a second. Any disagreements were resolved through discussion until consensus was reached.

## 3. Results

### 3.1. Studies retrieved

**Figure 1.**
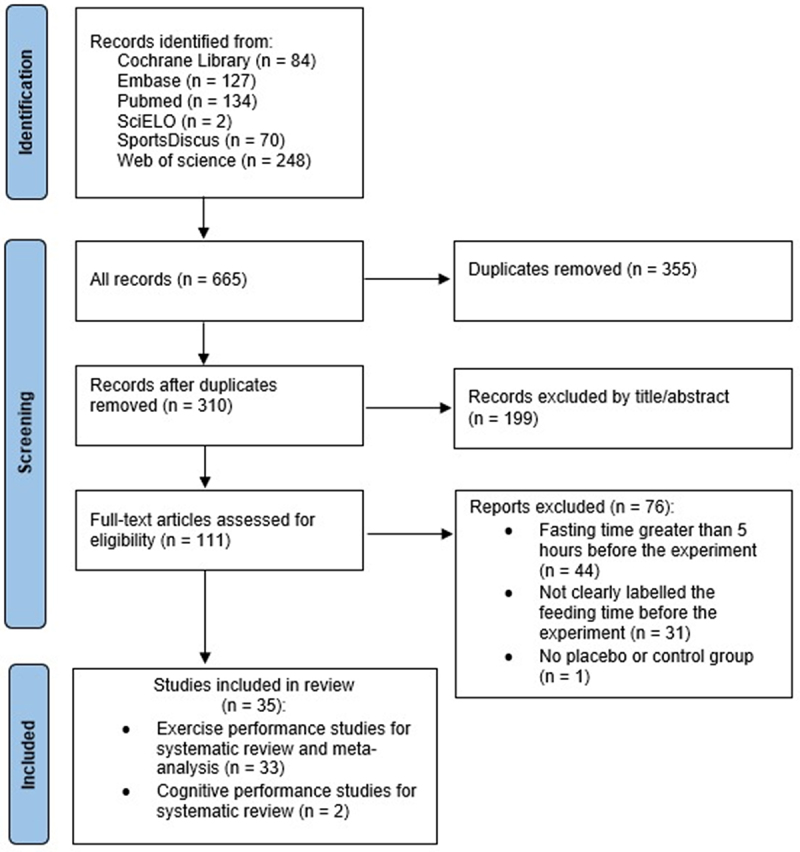
PRISMA flow diagram for included and excluded studies.

The initial search yielded 665 publications. After screening, a total of 35 studies met the criteria (Figure 1). These studies provided 76 effect size estimates (k = 76), of which 33 studies (k = 73) examined exercise performance and 2 studies (k = 3) examined cognitive performance. Due to the limited number of studies on cognitive performance, the subsequent summaries and statistical analyses focus exclusively on exercise performance. For further details on cognitive performance, please refer to Table 1.

**Table 1.** Summary and results of the studies reviewed assessing the effect of carbohydrate mouth rinsing under fed states on cognitive performance.

Study; Design	Cognitive protocol	Sample (n)	Training status	Fed time (h)	Meal contents	Mouth rinse protocol	Washout period	Performance outcomes	Statistical significance
Konishi et al. [53]; NS Crossover	Cognitive: Stroop test	4 males and 4 females	Young healthy	3 before	Breakfast (485 kcal: 75.6 gCHO, 18.8 g PRO, 10.1 g fat)	6.4% MD and water for 5 s	Min 5 days	Pre- and post- reaction time (ms): MD: 531 ± 54 and 522 ± 80 vs PLA: 529 ± 45 and 547 ± 60	Yes ↓ (*p* = 0.028)
Pomportes et al. [54]; SB Crossover	Cognitive: Duration-production task (DPT) and Simon task (ST)	16 males and 6 females	Physically active (train 3–8 h/week)	3.5 before	NS	7% CHO (89% fructose +11% MD) and orangesugarless syrup for 20 s	Min 72 h	Mean time of DPT (ms): CHO: 1285.9 ± 119 vs PLA: 1333.2 ± 155.8; Mean reaction time of ST (ms): 345.6 ± 17.1 vs PLA: 346.2 ± 17.6	DPT: Yes ↓ (*p* < 0.05) ST: No (*p* > 0.05)

***CHO***: Carbohydrate; ***SB***: Single blind; ***g***: Gram; ***h***: Hour; ***kcal***: Kilocalories; ***MD***: Maltodextrin; ***ms***: Millisecond; ***NS***: Not suitable/Not specified; ***n***: Sample size; ***PLA***: Placebo; ***PRO***: Protein; ***↓***, represents significantly decreased.

### 3.2. Characteristics of included studies

Across all studies, a total of 444 participants were included (380 males, 64 females), with sample sizes ranging from 7 to 33. The majority of exercise performance studies recruited only males (*n* = 31, k = 69), with very limited female or mixed-gender samples. Most participants were trained individuals (*n* = 30, k = 67), with varying training levels from recreational to elite. Only three studies (k = 6) involved untrained participants.

Rinse duration ranged from 5 to 15 seconds, with most studies using 5 seconds (*n* = 13, k = 27) or 10 s (*n* = 16, k = 35). The most common CHO concentration was 6.4% (*n* = 16, k = 39), while other solutions ranged from 4% to 16%. Maltodextrin was the predominant CHO source (*n* = 27, k = 56), followed by glucose (*n* = 4, k = 7) and sucrose (*n* = 3, k = 10).

Based on the exercise modality, 25 studies (k = 47) focused on aerobic capacity, while 12 studies (k = 26) examined anaerobic outcomes such as strength and power. Regarding pre-exercise nutrition, 13 studies (k = 20) involved high-CHO meals (≥60% CHO), 8 studies (k = 20) involved medium-CHO meals, and 2 studies (k = 6) involved low-CHO intake. Ten studies did not specify dietary content (k = 27). For more details, please refer to Tables 2 and 3.

**Table 2.** Summary and results of the studies reviewed assessing the effect of carbohydrate mouth rinsing under fed states on exercise performance.

Study; Design	Exercise protocol	Sample (n)	Training status	Fed time (h)	Meal contents	Mouth rinse protocol	Washout period	Performance outcomes	Statistical significance
Azevedo et al. [55]; DB Crossover	Normal or fast or slow ~60-min Running TT	9 males	Older runner (200 ± 60 km/week)	~0.33 before	Standardized breakfast (Banana, whole wheat bread with ham and cheese, grape juice)	6% MD and artificial sweetener for 10 s	7 days	Velocity (km): Normal: MD: 14 ± 2.21 vs PLA: 13.64 ± 1.98; Fast: MD: 13.61 ± 1.74 vs PLA: 13.91 ± 1.82; Slow: MD: 13.71 ± 2.08 vs PLA: 12.92 ± 2.6	No (*p* > 0.05)
Baltazar-Martins & Del Coso [56]; DB Crossover	Cycling TT	16 males	Well-trained cyclists	3 before	Pre-competition meal (at least 3 g/kg of body mass of CHO)	6.4% MD and artificial sweetener for 5 s	7 days	Mean power output (W): MD: 231 ± 46 vs PLA: 222 ± 51	Yes ↑ (*p* = 0.04)
Bastos-Silva et al. [57]; DB Crossover	Moderate (MIE) and high-intensity (HIE) cycling TTE	13 males	physically active (Min 150 min·week^−1^ of moderate exercise)	2 before	NS	6.4% MD and orange juice for 10 s	Min 72 h Max 96 h	Mean time: MIE (min): MD: 76.6 ± 19.7 vs PLA: 65.4 ± 15.2; HIE (s): MD: 177.3 ± 42.2 vs PLA: 163.0 ± 26.7	MIE: Yes ↑ (*p* = 0.01) HIE: No (*p* = 0.1)
Bastos-Silva et al. [58]; SB Crossover	Cycling sprint TTE	14 males	Physically active	2 before	NS	6.4% MD and juice for 10 s	Min 72 h	Mean time (s): MD: 174.3 ± 42.8 vs PLA 166.7 ± 26.3; Energy contribution (KJ): MD: 203.2 ± 46.4 vs PLA: 196.5 ± 45.2	No (*p* > 0.05)
Bastos-Silva et al. [59]; DB Crossover	Resistance exercise: 80% 1RM leg press (LP) and bench press (BP)	12 males	Resistance training for 2 more years	2 before	NS	6.4% MD and juice for 10 s	72 h	Repetitions (LP): MD: 13.5 ± 4.8 vs PLA 11.5 ± 4.4 vs CON: 12.4 ± 4.4; (BP): MD: 8.2 ± 1.6 vs PLA: 7.1 ± 2.4 vs CON: 6.8 ± 1.8; Training load volume (kg) (LP): MD: 2006.7 ± 825.2 vs PLA: 1712.5 ± 772.9 vs CON: 1817.1 ± 672.6; (BP): MD: 557.1 ± 155.4 vs PLA: 495.9 ± 206.1 vs CON: 476.1 ± 173.3	LP: No (*p* > 0.05) BP: Yes MD vs CON only ↑ (*p* < 0.05)
Beelen et al. [60]; DB Crossover	1 h cycling TT	14 males	Competitive cyclists (trained twice/week)	~2 before	Standardized breakfast (39.5 kJ/kg; 67% CHO, 13% PRO, 20% fat)	6.4% MD and water for 5 s	Min 7 days	Mean time (min): MD: 68.14 ± 4.27 vs PLA: 67.52 ± 3.74; mean power output (W): MD: 265 ± 18.71 vs PLA: 266 ± 18.71	No (*p* > 0.05)
Black et al. [61]; DB Crossover	Knee maximal voluntary isometric strength (MVC)	6 males and 7 females	Recreationally active	~2 before	Light, mixed (∼50% CHO, 20% PRO and 30% fat)	8% MD and artificial sweetener for 20 s	48–96 h	Change from pre- and post-MVC (N·m): MD: 17.22 ± 7.5 vs PLA: 18.69 ± 10.02	Yes ↓, only with Post vs Pre (*p* ≤ 0.05)
Black et al. [62]; DB Crossover	Knee maximal voluntary isometric strength (MVC)	12 males	Physically active	2 before	Light, mixed (∼50% CHO, 20% PRO and 30% fat)	8% MD and artificial sweetener for 20 s	Min 48 h	Change from pre- and post-MVC (N.m): After 20% MVC training exhaustion: MD: 22.53 ± 30.08 vs PLA: 27.82 ± 56.7; After 80% MVC training exhaustion: MD: 12.4 ± 28.25 vs PLA: 9.16 ± 21.56	Yes ↓, only with Post vs Pre (*p* < 0.05)
Chiu et al. [63] SB Crossover	Physical fitness test and simulation test for Taekwondo specialty	13 males	Trained taekwondo athletes (≥6 years of experience)	~3 before	Standard lunch (829 kcal: 50% CHO, 20% PRO, 30% fat)	6.4% MD and mineral water for 15 s	7–10 days	Jump height (cm): 1^st^: MD: 40.26 ± 5.12 vs PLA: 41.03 ± 6.92; 2^nd^: MD: 38.29 ± 4.79 vs PLA: 37.52 ± 5.81; Frequency speed of kick test: 1^st^: MD: 114.1 ± 53 vs PLA: 107.1 ± 51.3; 2^nd^: MD: 101.3 ± 46.5 vs PLA: 94.1 ± 45.5; Mean power output of 3 rounds 5-s cycling sprints (W): MD: 357.1 ± 90.34 vs PLA: 367.13 ± 123.53; Peak power output of 3 rounds 5-s cycling sprints (W): MD: 413.71 ± 99.34 vs PLA: 416.67 ± 130.54	No (*p* > 0.05)
Cramer et al. [64]; DB Crossover	40-km cycling TT	8 males	Well-trained cyclists (Min 250 km/week)	3 before	NS	6.5% MD and sugar-free cordial/water mixture for 5 s	5–7 days	Mean time (min): MD: 63.9 ± 3.2 vs PLA: 64.3 ± 2.8; Mean power output (W): MD: 251 ± 23 vs PLA: 242 ± 18	No (*p >* 0.05)
Devenney et al. [65]; DB Crossover	Cycling TT	12 males	Recreationally active	2 ~ 3 before	49% CHO, 18% PRO, 33% fat	0 or 6 or 16% MD for 5 s	NS	Mean time (min): MD 16%: 57.9 ± 7.6 vs 6%: 58.8 ± 7 vs PLA: 62.3 ± 7.6; Mean power output (W): MD 16%: 177 ± 23 vs 6% :174 ± 20 vs PLA: 163 ± 23	Yes ↑ MD 6% and 16% vs PLA (*p* < 0.05)
Devenney et al. [66]; DB Crossover	HIIT TTE (running)	8 males	Recreationally trained (5–8 h/week)	2–3 before	49% CHO, 18% PRO, 33% fat	6% MD and orange juice for 5 s	NS	Distance (m): MD: 5127 ± 1367 vs PLA: 4535 ± 1217	No (*p* = 0.218)
Durkin et al. [67]; SB Crossover	Resistance exercise: 40% 1RM bench press (BP) and squat lifts (SL) 6 sets to failure	12 males	Resistance training for 2 more years	2 before	Low-carbohydrate breakfast (267 kcal, 14.6 g CHO, 21.7 g PRO, 13.9 g fat)	6.4% MD and artificial sweetener for 10 s	NS	Total volume workload (kg): MD: 9354 ± 7104.4 vs PLA: 8525 ± 6619.7; Repetitions (BP): MD: 120 ± 83.1 vs PLA: 115 ± 76.2; (SL): MD: 107 ± 90.1 vs PLA: 92 ± 55.4	SL and Total volume workload: Yes ↑ (*p* < 0.05) BP: No (*p* = 0.146)
Faezeh & Zahra (2020) [68]; DB parallel	Ramp running TTE; Resistance exercise: Chest press (CP), Leg press (LP)	33 females	Sedentary and overweight (train less than 90 min/week)	2 before	Low-fat breakfast (300–500 kcal)	6% MD and water for 10 s	Min 72 h	Change from pre- and post-TTE (s): MD: 38.3 ± 33 vs PLA: 4.1 ± 55; Change from pre- and post-CP: MD: 6.1 ± 6 vs PLA: 4.6 ± 10.7; Change pre- and post-LP: MD: 15.5 ± 14 vs PLA: 9 ± 13	TTE: Yes ↑ (*p =* 0.03) CP and LP: No (*p >* 0.05)
Ferreira et al. [69]; DB Crossover	30-Km cycling TT	11 males	Trained cyclists (Min 5 h/week)	2–3 before	Mean ~644 kcal with ~68.4 g CHO	6.4% MD and artificial sweetener for 10 s	NS	Mean time (min): MD: 54.5 ± 2.9 vs PLA: 54.7 ± 2.9 vs CON: 54.5 ± 2.5; Mean power output (W): MD: 198.6 ± 25.9 vs PLA: 196.6 ± 26.9 vs CON: 196.9 ± 22.4	No (*p* > 0.05)
Gam et al. [70]; SB Crossover	1000-KJ cycling TT	10 males	Moderately trained cyclists	4 before	NS	6.4% MD and water for 5 s	7 days	Mean time (min): MD: 65.7 ± 11.07 vs PLA: 69.4 ± 13.81 vs CON: 67.6 ± 12.68	Yes ↓ (*p* < 0.05)
Gough et al. [71]; DB Crossover	Repeated sprint during simulated soccer match play	9 males	Experienced recreational soccer player	~2 before	High-carbohydrate meal (2 g·kg^−1^body mass)	10% MD and artificial sweetener for 10 s	Min 96 h	Change from pre- and post-mean power output (W): MD: 0.68 ± 24.61 vs PLA: 9.2 ± 28.52; Change from pre- and post-peak power output (W): MD: 2.46 ± 21.98 vs PLA: 9.16 ± 25.57	No (*p* > 0.05)
Ispoglou et al. [72]; DB Crossover	1 h cycling TT	9 males	Cyclists (min trained 3 times/week)	~3 before (afternoon)	Typical pre-race diet (NS)	0 or 4 or 6 or 8% CHO (89% sucrose and 11% glucose) for 5 s	7 days	Mean time (min): CHO 8%: 63 ± 4 vs 6%: 63.4 ± 3.4 vs 4%: 62.8 ± 4 vs PLA: 62 ± 3; Mean power output (W): CHO 8%: 247 ± 33 vs 6% :246 ± 31 vs 248 ± 28 vs PLA: 251 ± 28	No (*p* > 0.05)
Jensen et al. [73]; DB Crossover	~30-min cycling TT	10 males	Well-trained cyclists	First refill 4 before + continuous refill 2 before	Standardized pre-trial snack (158 g CHO, 18 g protein) + 1500 mL 4% CHO solution	8% MD and artificial sweeter for 5–10 s		Mean time (s): MD: 1893.84 ± 26 vs PLA: 1928.82 ± 46.5	No (*p* = 0.51)
Kamaruddin et al. [74]; DB Crossover	Running TTE under dehydrated (DE) or euhydrated (EU)	12 males	Endurance trained runners (train 5 ± 1/week)	~2 before	Standardized breakfast (2.5 g.kg^−1^ of body mass calories)	6% glucose and artificial sweeter for 10 s	7 days	TTE (min): DE: CHO: 80.2 ± 4 vs PLA: 76.1 ± 3.8; EU: CHO: 77.8 ± 3.7vs PLA: 75.9 ± 4	Yes (*p* < 0.05)
Kamaruddin et al. [75]; DB Crossover	Running TTE	12 males	Endurance runners (train 5 ± 1/week)	~2 before	Standardized breakfast (2.5 g.kg^−1^ of body mass calories)	6% glucose and artificial sweeter for 10 s	7 days	TTE (min): CHO: 54.7 ± 5.4 vs PLA: 53.6 ± 5.1 vs CON: 48.4 ± 3.6	Yes, only with CHO and PLA vs CON ↑ (*p* < 0.05)
Luden et al. [76]; DB Crossover	2-km TT; Maximum voluntary contract strength (MVC)	8 males	Endurance-trained cyclists (≥3 days of cycling/week)	2 before	Standardized breakfast (500 Kacl: 90–100 g CHO, 8–12 g PRO, 4–8 g fat)	6.4% MD and deionized water for 5 s	7–10 days	Mean time of TT (s): MD: 192.4 ± 8.2 vs PLA: 200.1 ± 10.8; Change from per- and post-MVC (N): MD: 25 ± 25 vs PLA:34 ± 40	No (*p* > 0.05)
Phillips et al. [77]; DB Crossover	30s cycling sprint	12 males	Physically active	2 before	Controlled Breakfast (NS)	6% MD and berry flavoring for 5 s	3–7 days	Peak power output (W): MD: 13.51 ± 2.19 vs PLA: 13.20 ± 2.14	Yes ↑ (*p* < 0.05)
Pires et al. [78]; DB Crossover	4-Km TT	9 males	Recreational cyclists	2 before	Standard breakfast (NS)	6.4% glucose and artificial sweetener for 10 s	NS	Mean time (s): CHO: 386.4 ± 28 vs PLA: 385.4 ± 22.4; Mean power output (W): CHO: 275.4 ± 43.3 vs PLA: 277 ± 35	No (*p* > 0.05)
Pires et al. [79]; DB Crossover	MIT TTE (cycling)	9 males	Recreational cyclists	2 before	Individualized breakfast (~55% CHO, ~25% PRO, ~20% fat)	6.4% glucose and artificial sweetener for 10 s	3–7 days	Mean time (s): CHO: 610.4 ± 99.1 vs PLA: 622.4 ± 94.8; Peak power output (W): CHO: 328.8 ± 42 vs PLA: 332.3 ± 42.2	No (*p* > 0.05)
Pottier et al. [80]; DB Crossover	~1 h cycling TT	12 males	triathletes	Min 3 before	CHO-rich but low-fat diets	6% isotonic carbohydrate electrolyte solution (CES) with 5.4 g sucrose and 0.46 g glucose, and artificial sweetener for 5 s	Min 48 h	Time (min) CES: 61.7 ± 5.1 vs PLA: 64.1 ± 6.5; Mean power output (W): CES: 265 ± 26.4 vs PLA: 256.5 ± 30.8	Yes ↑ *(p* = 0.02)
Rollo et al. [81]; DB Crossover	Jogging and running sprints	10 males	Amateur soccer players	3 before	Usual match-day meal	10% MD and sweetener for 10 s	7 days	Mean jogging speed (km·h^−1^): MD: 11.3 ± 0.7 vs PLA: 10.5 ± 1.3; 15-m sprint speed (km·h^−1^): MD: 20.4 ± 1 vs PLA: 20.1 ± 1.2	Jogging speed Yes ↑ (*p* = 0.01) 15-m sprint speed: No (*p* = 0.316)
Sinclair et al. [82]; NS Crossover	~30-min cycling TT	11 males	Recreational cyclists	4 before	NS	6.4% MD and water for 5 s and 10 s	7 days	Distance (km): 10 s MD: 20.4 ± 2.3 vs PLA: 19.2 ± 2.2; Mean speed (km/h^−1^): 5 s MD: 37.95 ± 3.95 vs 10 s MD: 38.66 ± 4.13 vs PLA: 36.06 ± 4.40; Power output (W): 5 s MD: 152.35 ± 17.42 vs 10 s MD: 155.63 ± 17.05 vs PLA: 145.73 ± 13.55	Distance: Yes ↑ (*p* < 0.05); Mean speed and power output: Yes, with 10 s MD vs PLA only ↑ (*p* < 0.05)
Whitham & McKinney [83]; DB Crossover	1 h running TT	7 males	Recreationally active	4 before	816kcal standardized breakfast (carbohydrate 77.6%, fat 13.8%, protein 8.7%)	6% MD and lemon juice for 5 s	Min 5 days	Mean distance (m): MD: 9333 ± 988 vs PLA: 9309 ± 993	No (*p* = 0.933)

***CHO***: Carbohydrate; ***cm***: Centimeter; ***DB***: Double blind; ***g***: Gram; ***h***: Hour; ***kcal***: Kilocalories; ***km***: Kilometer; ***KJ***: Kilojoules; ***MD***: Maltodextrin; ***m***: Meter; ***min***: Minutes; ***Min***: Minimal; ***N***:Newton; ***NS***: Not suitable/Not specified; ***n***: Sample size; ***PLA***: Placebo; ***PRO***: Protein; ***TT***: Time trail; ***TTE***: Time to exhaustion; ***↑***, represents significantly improved; ***↓***, represents significantly decreased.

**Table 3.** Summary and results of the studies reviewed directly assessing the effect of carbohydrate mouth rinsing between fasted and fed state on exercise performance.

Study; Design	Exercise/cognitive protocol	Sample (n)	Training status	Fed time/ Fast time (h)	Meal contents	Mouth rinse protocol	Washout period	Performance outcomes	Statistical significance
Ataide-Silva et al. [14]; DB Crossover	20-Km cycling TT	8 males	Healthy and physically active	2 before/ Overnight (12); Exercise-depleted muscle glycogen (DEP) + Overnight (12)	Breakfast (485 ± 277 kcal, 63.6% CHO, 11.7% PRO, 24.7% fat)	6.4% MD and water for 10 s	Min 72 h, Max 7 days	Mean times (min): Fed: MD: 40.88 ± 1.68 vs PLA: 40.65 ± 1.43; Fast: MD: 41.76 ± 1.26 vs PLA: 42.97 ± 1.7; DEP-Fast: MD: 44.69 ± 1.38 vs PLA: 48 ± 2.05	Yes↓ only under DEP-Fast (*p* < 0.05)
Fares & Kayser [84]; NS Crossover	Cycling TTE	13 males	Nonathletic male	3 before/ Overnight (NS)	Standardized carbohydrate-rich breakfast (including CHO, PRO and small amount of fat)	6.4% MD and water for 5–10 s	Min 72 h Max 96 h	Mean time (min): Fed: MD: 56.6 ± 12.2 vs PLA: 54.7 ± 11.3; Fast: MD: 53.9 ± 12.8 vs PLA: 48.3 ± 15.3	MD vs PLA: Yes ↑ (*p* = 0.02) Fed vs Fast: No (*p* = 0.22)
Lane et al. [85]; DB Crossover	1 h cycling TT	12 males	Competitive endurance trained cyclists or triathletes	2 before/Overnight 9–10	Cereals, milk, fruits and fruit juices with 2.5 g-kg-1 of body mass CHO	10% MD and artificial sweetener for 10 s	~7 days	Mean power output (W) Fed: MD: 286 ± 20.8 vs PLA: 281 ± 17.3; Fast: MD: 282 ± 20.8 vs PLA: 273 ± 20.8	MD vs PLA: Yes ↑ (*p* < 0.02) Fed vs Fast: Yes, with only under PLA condition↑ (*p* < 0.01)
Trommelen et al. [10]; DB Crossover	~1 h cycling TT	14 males	Trained cyclists or triathletes (trained 4 times/week)	2 before/ Overnight (NS)	Standardized breakfast (36 ± 2 kJ/kg: 65 ± 7% carbohydrate, 17 ± 3% PRO, and 18 ± 4% fat)	6.4% sucrose and artificial sweeter for 5 s	Min 7 days	Mean time (min): Fed: MD: 69 ± 6.3 vs PLA: 67.6 ± 6.6; Fast: MD: 69.6 ± 7.5 vs PLA: 68.6 ± 7.2; Mean power output (W): Fed: MD: 253 ± 41 vs PLA: 258 ± 45; Fast: MD: 252 ± 46 vs PLA: 258 ± 45	No (*p* > 0.05)

***CHO***: Carbohydrate; ***DB***: Double blind; ***g***: Gram; ***h***: Hour; ***kcal***: Kilocalories; ***MD***: Maltodextrin; ***min***: Minutes; ***Min***: Minimal; ***NS***: Not suitable/Not specified; ***n***: Sample size; ***PLA***: Placebo; ***PRO***: Protein; ***s***: Seconds; ***TT***: Time trail; ***TTE***: Time to exhaustion; ***W***: Watt; ***↑***, represents significantly improved; ***↓***, represents significantly decreased.

### 3.3. Primary analysis

The meta-analysis revealed a small but significant ergogenic effect of CHO mouth rinse on exercise performance under fed conditions (k = 73, g = 0.18, 95% CI [0.09, 0.28], *p* < 0.01), with substantial heterogeneity (I^2^ = 65%). The prediction interval ranged from −0.30 to 0.66, indicating variability in potential true effects. Variance decomposition showed that 65.8% of the variance was due to between-study differences, justifying further moderator analyses [86]. The certainty of evidence for the overall effect was rated as low due to publication bias and inconsistency.

### 3.4. Moderator analysis

Moderator analysis revealed that exercise type significantly moderated the effect of CHO mouth rinse on performance (F (1, 71) = 7.68, *p* = 0.01), with greater benefits observed in aerobic exercise (g = 0.26, *p* < 0.05; low certainty) compared to anaerobic modalities (g = 0.02, *p* > 0.10; low certainty). Other moderators, including sex, training status, mouth rinse solution, rinse duration, and dietary composition, did not show statistically significant between-group effects (*p* > 0.10). However, moderator analyses revealed significant within-group effects in males (g = 0.19, *p* < 0.05; low certainty) and trained individuals (g = 0.17, *p* < 0.05; low certainty). Among rinse duration subgroups, the 5-second rinse showed a robust effect (g = 0.25, *p* < 0.01; moderate certainty), whereas longer rinses yielded weaker and less certain results. When categorized by CHO type, maltodextrin demonstrated a small but significant benefit (g = 0.21, *p* < 0.05; low certainty), while glucose and sucrose showed weaker and less reliable effects (very low to low certainty). Notably, not only did high-CHO meal conditions show significant ergogenic benefits (g = 0.20, *p* = 0.02; low certainty), but studies with unclear pre-exercise dietary content also reported significant improvements (g = 0.25, *p* = 0.01) although the certainty of evidence was rated as very low due to methodological limitations. For more information, please refer to Table 4.

**Table 4.** Summary data of moderator analysis.

Moderating variables	K	Hedges’ *g*	95% CI	*P*_*within*_	*I*^2^	*P*_*between*_	*GRADE*	*Comparisons*
**Sex**					65.04%	0.33		Female > Male (*p* = 0.40) Female > Mix (*p* = 0.15) Male > Mix (*p* = 0.23)
Male	69	0.19	[0.09, 0.28]	< 0.01			Low	
Female	3	0.45	[−0.17, 1.08]	0.15			Very low	
Mixed	1	−0.16	[−0.72, 0.40]	0.57			Very low	
**Training Status**					65.47%	0.51		Untrained > Trained (*p* = 0.51)
Trained	67	0.17	[0.07, 0.27]	< 0.01			Low	
Untrained	6	0.29	[−0.05, 0.63]	0.09			Very low	
**Exercise Type**					63.98%	0.01		Aerobic > Anaerobic (*p* = 0.01) Aerobic > Strength (*p* = 0.04) Aerobic > Power (*p* = 0.01) Strength > Power (*p* = 0.54)
Aerobic	47	0.26	[0.15, 0.37]	< 0.01			Low	
Anaerobic	26	0.02	[−0.13, 0.17]	0.81			Low	
					64.00%	0.02		
Strength)	17	0.05	[−0.13, 0.23]	0.59			Low	
Power)	9	−0.01	[−0.20, 0.17]	0.88			Low	
**Mouthwash solutions**					62.00%	0.36		MD > Glucose (*p* = 0.93) Glucose > Sucrose (*p* = 0.29) MD > Sucrose (*p* = 0.15)
Maltodextrin	56	0.21	[0.10, 0.31]	< 0.01			Low	
Glucose	7	0.19	[−0.08, 0.46]	0.16			Very low	
Sucrose	10	−0.02	[−0.31, 0.27]	0.90			Low	
**Rinse time**					63.45%	0.13		(Rinse ≦ 10 s) > (Rinse ＞ 10 s) (*p* = 0.13) Rinse 10 s > (Rinse ＞ 10 s) (*p* = 0.12) Rinse 10 s > Rinse 5 s (*p* = 0.37) Rinse 5 to 10 s > Rinse 10 s (*p* = 0.23) Rinse 5 s > (Rinse ＞ 10 s) (*p* = 0.28) Rinse 5 to 10 s > Rinse 5 s (*p* = 0.12) Rinse 5 to 10 s > (Rinse ＞ 10 s) (*p* = 0.05)
Rinse > 10 s	9	−0.03	[−0.33, 0.26]	0.83			Very low	
Rinse ≦ 10 s	64	0.21	[0.11, 0.30]	< 0.01			Low	
					64.16%	0.64		
Rinse 5 s)	27	0.25	[0.01, 0.29]	0.04			Moderate	
Rinse 10 s)	35	0.22	[0.10, 0.35]	< 0.01			Low	
Rinse 5 to 10 s)	2	0.48	[0.07, 0.89]	0.02			Very low	
**Dietary contents**					65.17%	0.51		High > Medium (*p* = 0.29) Low > High (*p* = 0.78) Low > Medium (*p* = 0.39) Low > Unclear (*p* = 0.98) Unclear > High (*p* = 0.64) Unclear > Medium (*p* = 0.15)
High CHO intake	20	0.20	[0.04, 0.35]	0.02			Low	
Medium CHO intake	20	0.06	[−0.13, 0.25]	0.52			Low	
Low CHO intake	6	0.26	[−0.14, 0.65]	0.20			Very low	
Unclear CHO intake	27	0.25	[0.01, 0.42]	0.01			Very low	

*CHO*, carbohydrate; *MD*, maltodextrin; *K*, the total number of effects included in the pooled effect size; *Hedges’g*, the effect size indicators used in the pooled; *95% CI*, 95% confidence interval; *P-value*, statistically significant *p* values for pooled results; *within* represents the specific p-value of each category in the moderator variable, while *between* represents the p-value of the significant difference between each category; *I*^*2*^, quantitative indicators of heterogeneity; *Power*, statistical power for pooled effect size; *GRADE*, grading of recommendations assessment, development, and evaluation, a system for evaluating the quality of evidence and strength of recommendations, where levels one to four are classified as “very low,” “low,” “moderate,” and “high”.

**Figure 2.**
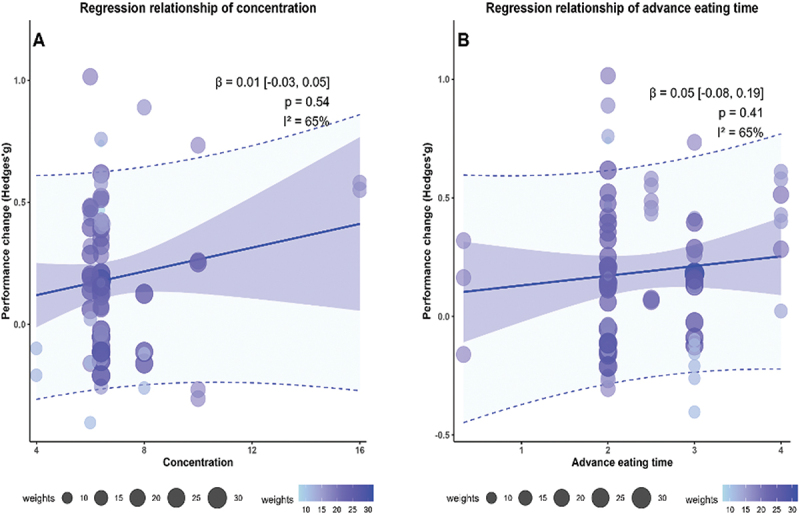
Meta-regression analysis: A. based on CHO mouth rinse concentration; B. based on advance eating time (*Notes*: *lines* represent effect size regression relationships; *shading* represents 95% confidence intervals; *dashed lines* represent the prediction intervals; *circles* represent different effect sizes.).

Continuous meta-regression analyses indicated no significant linear associations between CHO mouth rinsing concentration and performance outcomes (β = 0.01, 95% CI [−0.03, 0.05], *p* = 0.54; I^2^ = 65%, Figure 2A) or between pre-exercise meal timing and performance (β = 0.05, 95% CI [−0.08, 0.19], *p* = 0.41; I^2^ = 65%, Figure 2B). Cubic regression models were also explored to assess potential non-linear effects, but neither model improved fit over the linear alternatives (both ΔAIC < 0.3), and regression coefficients remained non-significant. These findings suggest that there is no clear or robust association between CHO mouth rinsing concentration or the timing of pre-exercise meals and exercise performance under fed conditions.

### 3.5. Subgroup analysis

**Figure 3.**
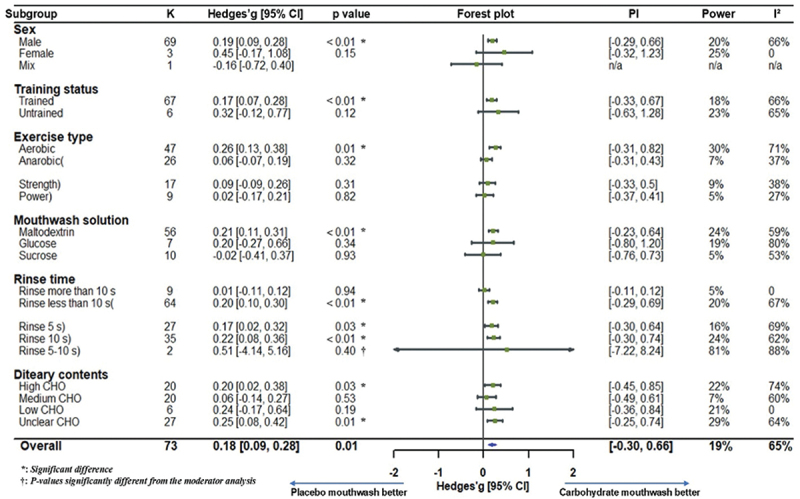
CHO mouth rinse subgroup analysis (*Notes*: *K*, the total number of effects included in the pooled effect size; *Hedges’g*, the effect size indicators used in the pooled; *95% CI*, 95% confidence interval; *PI*, prediction interval; *P-value*, statistically significant *p* values for pooled results; *I*^*2*^, quantitative indicators of heterogeneity; *Power*, statistical power for pooled effect size.).

In order to validate the effect of each moderator variable on the overall effect and to further explore the effect sizes within each categorization, we conducted conventional subgroup analyses under a three-level framework. Some discrepancies were noted between regression and subgroup results, likely due to smaller sample sizes within subgroups, which can increase heterogeneity and reduce statistical power [87,88]. For example, subgroup analysis of rinses lasting 5–10 seconds showed a large but statistically non-significant effect (g = 0.51, *p* = 0.40, I^2^ = 88%), suggesting instability in these subgroup estimates. For further details, please refer to Figure 3.

### 3.6. Risk of bias and certainty of the evidence

Most studies were judged as low risk of bias in outcome measurement and missing data. However, concerns were more frequent in the domains of randomization, deviations from intended interventions, and selective reporting, where several studies were rated as having some concerns or high risk. Overall, about half of the included trials were judged at low risk of bias, while the remainder raised some methodological concerns. Full domain-level assessments are provided in the Electronic Supplementary Material Appendix S2.

Publication bias was assessed using funnel plots and Egger’s regression tests within a three-level meta-analytic framework. For the overall effect of exercise performance, a significant association between effect size and standard error was detected (*p* = 0.02), indicating potential publication bias or small-study effects. When the same tests were applied within subgroups (k ≥ 10), evidence of asymmetry was observed for male participants (*p* = 0.01), trained individuals (*p* = 0.03), aerobic exercise outcomes (*p* = 0.02), maltodextrin mouth rinse (*p* = 0.03), rinse duration ≤10 s (*p* = 0.04), and high-CHO meal conditions (*p* = 0.02). No significant asymmetry was detected in other subgroups. For more information, including funnel plots, statistical power analyses, and reproducibility indices (R-index = 0–19.2%), please refer to the Electronic Supplementary Material Appendices S3 and S4.

Overall, the certainty of evidence across most subgroup analyses was rated as low to very low, primarily due to the risk of bias, inconsistency, and imprecision (Electronic Supplementary Material Appendix S5). The only exception was the rinse duration of 5 seconds, which reached a moderate level of certainty. For cognitive performance, assessed using a narrative-adapted GRADE approach, the certainty was judged as very low, reflecting the limited number of studies and methodological constraints. These findings highlight that while some ergogenic effects of CHO mouth rinsing are supported, the overall strength of evidence remains limited and warrants cautious interpretation.

### 3.7. Sensitivity analysis

Sensitivity testing using different assumed correlation coefficients (*r* = 0.2, 0.5) revealed that lower values slightly reduced the pooled g and decreased heterogeneity (Table 5) but did not alter the significance of the findings.

**Table 5.** Summary of sensitivity analysis results.

Test Methods	K	Hedges’ *g*	95% CI	*P*	PI	*Power*	*I*^2^
r = 0.85	73	0.18	[0.09, 0.28]	< 0.01	[−0.30, 0.66]	19%	65%
r = 0.50	73	0.12	[0.03, 0.22]	< 0.01	[−0.17, 0.42]	7%	4%
r = 0.20	73	0.13	[0.04, 0.22]	< 0.01	[−0.05, 0.31]	7%	0
Remove outliers	69	0.16	[0.07, 0.24]	< 0.01	[−0.25, 0.57]	16%	56%
MLE	73	0.18	[0.09, 0.28]	< 0.01	[−0.29, 0.65]	19%	65%

K, the total number of effects included in the pooled effect size; *Hedges’g*, the effect size indicators used in the pooled; *95%CI*, 95% confidence interval; 95%PI, Prediction interval, *P-value*, statistically significant *p* values for pooled results; *I*^*2*^, quantitative indicators of heterogeneity; *PI*, prediction interval; *Power*, statistical power for pooled effect size; *r*, correlation coefficient between carbohydrates group and placebo group; *MLE*, maximum likelihood estimation model.

Leave-one-out analysis showed pooled effects remained stable across exclusions (g range: 0.17–0.20; I^2^ : 52–60%; all *p* < 0.01), indicating robustness of the results (Electronic Supplementary Material Appendix S6).

Outlier diagnostics using Cook’s distance and studentized residuals identified several studies as influential. Between-study outliers included Black et al. (2018), Jensen et al. (2018), and Luden et al. (2016); within-study outliers included Kamaruddin et al. (2019) and Rollo et al. (2015) were found to have intra-study outliers [61,73,74,76,81]. Excluding these studies resulted in a slightly reduced but still significant pooled effect (k = 67, g = 0.16, *p* < 0.01), confirming the robustness of the primary findings (Table 5).

## 4. Discussion

Although CHO mouth rinsing has been widely studied for its ergogenic potential, the majority of investigations have focused on fasted-state protocols. In contrast, athletes typically train and compete in the fed state, making it essential to evaluate whether CHO mouth rinsing retains its efficacy under these real-world conditions. To our knowledge, this is the first systematic review and meta-analysis to synthesize fed-state evidence across both exercise and cognitive outcomes. The key findings indicate that postprandial CHO mouth rinsing has the potential to enhance not only physical performance but also cognitive function, thereby broadening its practical relevance for sport.

### 4.1. Cognitive performance

In many sports, optimal outcomes depend not only on physical capacity but also on the ability to perform complex cognitive tasks under competitive pressure, such as decision-making, attentional control, and rapid reaction [89]. CHO mouth rinsing offers a unique, non-metabolic pathway for performance enhancement by stimulating oral receptors and activating cortical regions implicated in cognitive control, such as the anterior cingulate cortex and the dorsolateral prefrontal cortex [12,13,54].

While numerous studies have examined the physical ergogenic effects of CHO mouth rinsing, its impact on cognition, particularly under fed conditions, remains underexplored. To our knowledge, no prior systematic review or meta-analysis has synthesized evidence in this area. In our review, only two studies met the eligibility criteria for cognitive outcomes. Konishi et al. (2017) study recruited 8 subjects (mixed gender) [53], who performed the Stroop cognitive test 3 hours after a meal and were exposed to a 6.4% maltodextrin mouth rinsing for 5 seconds per trial. Pomportes et al. (2017) study recruited 24 subjects (mixed gender) who performed the Duration-Production and Simon cognitive tests, with intervention consisting of gargling a mixture of 7% fructose and maltodextrin for 20 seconds [54]. Despite the many differences between the two studies, especially in methodology, both studies suggested small, favorable effects of CHO mouth rinsing on cognition in the fed state. These findings align with those from Hosang et al. (2023), who observed similar benefits in fasted participants [90].

Although these early findings are promising, the certainty of evidence for cognitive outcomes was judged as very low due to the limited number of studies and variability in cognitive measures, which also precluded meta-analysis. Therefore, our conclusions should be interpreted cautiously. Future studies should standardize cognitive testing protocols, control pre-testing nutrition, and incorporate neurophysiological tools (e.g. EEG, fMRI) to deepen understanding of the cognitive effects of CHO mouth rinsing. Such research may support its use not only for physical but also mental performance enhancement in sport.

### 4.2. Exercise performance

Pre-exercise nutritional status is a recognized methodological determinant of CHO mouth rinse efficacy [6,9]. A substantial body of clinical studies [85,91–93] and meta-analyses [94,95] has demonstrated that CHO mouth rinsing can provide performance benefits, particularly under fasting conditions. However, in practical terms, most individuals applying CHO mouth rinsing may not necessarily be fasting. Controversial findings have emerged when evaluating exercise performance under various postprandial conditions, with some studies reporting ineffective [60,72,78,83] and others showing effective [80,84,85] results of CHO mouth rinsing. Our analyses synthesized available evidence, providing a clearer understanding of performance outcomes when CHO mouth rinse is applied postprandially.

#### 4.2.1. Characterization of participants

A marked sex imbalance exists in the current literature, with most fed-state trials recruiting only male participants. Only two studies, Black et al. (2014) and Faezeh M, Zahra A. (2020), included female or mixed-gender cohorts [61,68]. As a result, current evidence primarily supports small but consistent benefits in males, whereas findings in females remain inconclusive due to the paucity of data and low certainty of evidence and require further targeted investigation.

Similarly, an imbalance was also evident in training status, with most studies recruiting trained individuals. Trained participants demonstrated more consistent improvements, supported by low-certainty evidence, whereas untrained participants showed a comparable estimated effect size but with wide confidence intervals, resulting in very low certainty. Rather than indicating the absence of benefit, this uncertainty underscores the limitations of the current evidence base and highlights the need to consider potential physiological and methodological explanations for these divergent patterns.

Indeed, trained individuals may exhibit enhanced central and peripheral efficiency, including more sensitive oral – cerebral pathways, improved motor unit recruitment, and stronger corticospinal excitability, making them more responsive to centrally mediated stimuli like CHO mouth rinsing. In contrast, untrained individuals may be more constrained by peripheral fatigue or inconsistent pacing strategies, which could mask potential benefits.

Overall, these findings highlight the need for future trials to explicitly stratify subjects by sex and training status, and to apply standardized definitions of training levels such as those outlined by McKay et al. (2022) [40], to improve comparability. Furthermore, increasing the representation of female and novice populations is critical to ensuring the translational relevance of CHO mouth rinsing protocols across diverse athletic and recreational settings.

#### 4.2.2. Exercise protocol

Our meta-analysis indicated that CHO mouth rinsing enhanced aerobic capacity under fed conditions more consistently than anaerobic outcomes. Specifically, aerobic capacity showed a greater improvement than both explosive performance (*p* = 0.01) and strength performance (*p* = 0.04), whereas no significant difference was observed between explosive and strength performance (*p* = 0.54). These results align with Brietzke et al. (2019) conclusion that CHO mouth rinsing positively affects cycling time trials in the absence of a clear feeding/fasting condition and Rodrigues Oliveira-Silva et al. (2023) finding that CHO mouth rinsing does not enhance the maximal strength [95,96].

Most aerobic capacity studies included in this review assessed cycling time trials, which require sustained energy expenditure. In these trials, the use of multiple mouth rinses during the exercise period may have helped maintain performance by continuously stimulating oral CHO receptors and sustaining central drive. A similar pattern was observed for anaerobic outcomes. Of the studies assessing anaerobic capacity, only the Durkin et al. (2021) and Phillips et al. (2014) studies included multiple mouth rinses in the testing protocol, and only these two studies found that CHO mouth rinsing significantly improved anaerobic capacity relative to placebo [67,77]. This suggests that the more pronounced effects in aerobic capacity may be linked to the prolonged nature of aerobic exercise, which allows repeated oral stimulation to exert cumulative central and possibly peripheral benefits. Further research should determine whether increasing the frequency or timing precision of mouth rinses can enhance anaerobic performance under fed conditions, as current evidence in this area is limited.

#### 4.2.3. Mouth rinse protocol – carbohydrate categories

Our meta-analysis results demonstrated that maltodextrin mouth rinsing consistently improved athletic performance under fed conditions. This aligns with Hartley et al. (2022) conclusions, who reported similar benefits without explicitly distinguishing between fed and fasted protocols [94]. Glucose mouth rinses showed a comparable effect size to maltodextrin, but the wide confidence intervals and small number of trials led to very low certainty, reflecting serious imprecision rather than absence of benefit. By contrast, sucrose showed no meaningful effect, supported by low-certainty evidence.

Mechanistically, the ergogenic effects of CHO mouth rinses are thought to be mediated primarily through the activation of oral receptors, particularly the T1R2/T1R3 sweet taste receptors [12,13]. Jeukendrup et al. (2008) showed that both sweet (glucose) and non-sweet (maltodextrin) CHO can activate brain regions linked to reward and motor control, suggesting a central nervous system mechanism for performance enhancement [97]. Lapis et al. (2016) further demonstrated that blocking T1R2/T1R3 with lactitol prevented the perception of sweet substances like glucose, maltose, and sucralose, but not glucose oligomers [98]. This implies that glucose oligomers, including maltodextrin, may activate alternative oral pathways independent of classical sweet taste receptors.

As a glucose oligomer, maltodextrin’s distinct molecular structure and sensory profile may underlie its more consistent ergogenic effects compared with monosaccharides like glucose or disaccharides like sucrose. However, the small number of studies on glucose and sucrose, combined with high within-group heterogeneity, means that differences among CHO types should be interpreted with caution.

Future research should aim to elucidate the distinct sensory and neurophysiological pathways activated by various CHO types, and explore how these pathways may differentially influence exercise performance under various nutritional states.

#### 4.2.4. Mouth rinse protocol – rinse duration

Rinsing duration has long been proposed as a methodological factor influencing the ergogenic effect of CHO mouth rinsing [9], yet few studies have systematically investigated this variable under fed conditions [77,82,99,100]. Our analysis indicated that rinsing for >10 seconds did not yield meaningful benefits, consistent with Hartley et al. (2022) [94] and Rodrigues Oliveira-Silva et al. (2023) [95], who suggested a potential threshold beyond which benefits may diminish. Several mechanisms may account for these findings. Prolonged rinsing during exercise may disrupt attentional focus, as sustained oral activity can interfere with task concentration [70]. It may also cause respirator-locomotor desynchronization, reducing oxygen uptake during submaximal workloads and offsetting potential central nervous system benefits [101,102].

In contrast, rinsing for 10 seconds or less, including 5-, 10-second and combined 5–10 second durations, appeared more effective, with no significant difference among these shorter durations. This differs from Hartley et al. (2022), who reported a greater effect at 10 seconds than at 5 seconds [94]. The discrepancy may reflect the inclusion of fasted-state studies in their analysis, whereas our results focus solely on fed-state conditions, in which oral receptor sensitivity and neurocognitive responses may differ. Additionally, within fed-state work, Sinclair et al. (2014) also observed greater distance after 10-second versus 5-second rinsing in a 30-minute cycling trial, but the absence of key safeguards (e.g. familiarization session, double-blind design) limits confidence in that finding [82].

However, the precision of these findings remains limited. Heterogeneity was high in both the 5-second (I^2^ = 69%) and 10-second (I^2^ = 62%) subgroups. In three-level subgroup analysis, the 5–10 second category was no longer significant (g = 0.51, *p* = 0.40), likely due to the small sample size (k = 2) and statistical instability [88,89]. In contrast, meta-regression using the full dataset provided greater power and consistency [34,35,103]. Overall, the current evidence suggests that rinse durations of 10 seconds or less are a pragmatic choice under fed conditions, although the certainty of evidence remains low to moderate. Larger, well-controlled trials are needed to determine whether an optimal duration threshold exists and to better clarify any small differences between 5 and 10 seconds.

#### 4.2.5. Mouth rinse protocol – rinse concentration

Contrary to the assumption that higher CHO mouth rinse concentrations would produce greater ergogenic effects, our meta-regression found no significant linear relationship between concentration and performance improvement under fed conditions. This is consistent with Hartley et al. (2022), who suggested that increasing concentration beyond a certain point may yield little or no additional benefit [94].

Most included studies used a CHO concentration of approximately 6.4% (median = 6.4%, Interquartile Range = 6–8%; see Tables 2–3 and Figure 2A), and our analysis found no evidence of a dose – response effect. This suggests that concentrations around 6.4% may be sufficient to elicit ergogenic benefits in the fed state. Similar conclusions were reported by Hartley et al. (2022) and Rodrigues Oliveira-Silva et al. (2023) studies [94,95], who also found no clear advantage from higher concentrations. Two clinical studies further support this interpretation: James et al. (2017) observed improved performance in a fasted state with a 7% solution, but no additional gain at 14% [104], while Devenney et al. (2016) found that both 6% and 16% solutions enhanced cycling performance under fed conditions without significant differences between them [65].

Notably, the available data are skewed toward moderate concentrations, with only three studies testing 10% [71,81,85] and one testing 16% [65]. This limited range at higher concentrations reduces the statistical power to detect potential threshold or non-linear patterns, such as an inverted U-shaped relationship where very high concentrations might impair performance. Therefore, while current data support the sufficiency of ~6.4% CHO mouthwash under fed conditions, future studies should prioritize direct comparisons between moderate (6–8%) and high (≥10%) concentrations to determine whether additional benefits exist, as even minor additional gains could be meaningful in elite athletic contexts where marginal improvements are highly valued.

#### 4.2.6. Advanced eating time

Meta-regression results indicated that within the 2–4-hour window after eating, which encompassed the timing used in most included studies, variations in meal timing were not significantly associated with CHO mouth rinse efficacy on exercise performance. This finding is consistent with Hartley et al. (2022) and Rodrigues Oliveira-Silva et al. (2023), who similarly reported that fasting duration did not significantly influence CHO mouth rinse effects, even in studies extending beyond four hours of fasting [94,95].

The primary mechanism of CHO mouth rinsing involves activation of oral CHO receptors such as T1R2/T1R3, which stimulates brain reward regions including the insular cortex and anterior cingulate cortex, thereby enhancing exercise drive and delaying fatigue [12,13]. Because this pathway operates independently of gastrointestinal absorption and circulating glucose, it is plausible that meal timing within the 2–4 hour postprandial period has little influence on its efficacy. Furthermore, postprandial insulin levels typically peak within 30–60 minutes after eating and decline to a stable level by 2–4 hours [105,106], which may explain the absence of a linear association in our analysis.

However, with the exception of Azevedo et al. (2023) study, all included studies tested CHO mouth rinsing at least 2 hours after eating [55]. No study directly compared immediate postprandial application (e.g. < 1 hour after a meal) with later postprandial periods. This leaves unanswered whether proximity to peak insulin levels could transiently alter oral receptor sensitivity or central responses. Future research should compare immediate (30–60 minutes) versus delayed ( > 2 hours) postprandial CHO mouth rinsing to clarify whether meal timing closer to ingestion modifies its ergogenic effect.

#### 4.2.7. Dietary contents

The potential benefits of CHO mouth rinsing may be influenced by endogenous CHO availability, including liver and muscle glycogen stores [60]. Although its mechanism is independent of circulating glucose, a hyperglycemic state may create a more favorable metabolic environment. Under high-CHO dietary conditions, CHO mouth rinsing showed a more consistent ergogenic effect, though the certainty of evidence was rated as low. In contrast, when participants consumed moderate- or low-CHO diets, CHO mouth rinsing did not yield clear benefits, and the certainty of evidence was very low due to limited data and imprecision.

High-CHO diets are known to elevate blood glucose and increase glycogen reserves, providing abundant energy substrates for exercise [107,108]. In such conditions, central drive enhancements from CHO mouth rinsing are more likely to translate into measurable performance gains. Conversely, hypoglycemia can impair central nervous system function by restricting energy supply, thereby limiting exercise capacity [109,110]. Even if CHO mouth rinsing activates central mechanisms, these benefits may be attenuated in a hypoglycemic state.

Notably, trials that did not report pretest dietary composition also demonstrated apparent performance gains, though the certainty of this evidence was very low. This suggests that factors beyond advanced eating time and dietary composition, possibly including individual metabolic status, habitual diet, or exercise protocol, may contribute to performance improvements. Future studies should report detailed pre-exercise dietary intake to allow more precise subgroup analyses.

#### 4.2.8. Fed vs. Fasted

Although our primary meta-analysis indicates that CHO mouth rinsing can improve exercise performance in the fed state, this finding is not fully consistent with several clinical trials [60,72,78,83]. For instance, Beelen et al. (2009) found no improvement in cycling time-trial performance with CHO mouth rinsing when participants were fed [60], and Whitham & McKinney (2007) reported no enhancement in running distance during a 45-minute trial with maltodextrin mouth rinse in the fed state [83]. Furthermore, an fMRI study comparing oral sucrose stimulation after an overnight fast (12 hours) versus immediately after a 700-kcal liquid meal (219 g·L − 1; 22% CHO) showed greater activation of brain regions such as the insula in the fasted state [111], suggesting that CHO mouth rinse effects may be more pronounced when fasted.

While the fasted state may confer relatively larger gains due to heightened oral-cerebral sensitivity, this does not diminish the practical utility of CHO mouth rinsing under real-world, fed-state scenarios [9]. In competition or training, athletes are typically in a fed state, where pre-exercise nutrition already enhances substrate availability; in such contexts, CHO mouth rinsing may serve as an additional central stimulus, providing a “finishing touch” that yields small yet potentially meaningful performance gains.

Table 3 summarizes four studies that directly compared the efficacy of CHO mouth rinsing under both nutritional conditions, with mixed findings [10,14,84,85]. Among these studies, Lane et al. (2013) observed the largest performance improvement when CHO mouth rinsing was combined with a CHO-rich pre-exercise meal during a one-hour simulated cycling time trial [85]. In contrast, Ataide-Silva et al. (2016) reported a significant benefit only after glycogen-depleting exercise followed by overnight fasting, but not in standard fasted or fed conditions [14]. Fares & Kayser (2011) found performance gains regardless of nutritional state during cycling to exhaustion at 60% of maximal power output [84], whereas Trommelen et al. (2015) reported no benefits in either state during a one-hour cycling trial [10].

Notably, since the publication of the Ataide-Silva study in 2016 [14], few if any new investigations have systematically examined the impact of nutritional status on CHO mouth rinsing efficacy. This highlights a critical gap in the literature. Future studies should prioritize exploring how test protocols, participant characteristics (e.g. training status, habitual diet), and feeding strategies interact with CHO mouthwash use. Such research will be essential to clarify whether combining CHO mouth rinsing with specific nutritional conditions can consistently optimize performance outcomes.

### 4.3. Mechanisms of action: central vs. Peripheral pathways

The ergogenic effects of CHO mouth rinsing have been predominantly attributed to central neural mechanisms involving oropharyngeal receptor activation [6,9]. However, given that our analysis focuses specifically on fed-state conditions, it is important to consider whether alternative pathways might contribute to the observed performance benefits, particularly when endogenous CHO availability is already elevated.

From a theoretical perspective, the oral cavity possesses absorptive capacity through sublingual and buccal mucosa [14], which could potentially allow small amounts of CHO to enter systemic circulation without gastrointestinal processing. Although blood glucose and insulin are typically elevated in the fed state [107,108], even very small amounts of CHO absorbed through the oral mucosa could, in theory, provide supplementary metabolic support. This sublingual route has been well documented for pharmaceutical delivery and represents a plausible complementary mechanism to receptor-mediated signaling [112,113].

However, several factors suggest that mucosal absorption is unlikely to contribute meaningfully to the observed effects. The brief exposure time during mouth rinsing (typically 5–15 seconds), combined with the limited absorptive capacity of the oral cavity, reduces the plausibility of efficient CHO uptake. Moreover, the standard protocol requires participants to expectorate the solution, a design feature intended to isolate central effects from metabolic influences.

These theoretical considerations are consistent with experimental evidence from both fasted and fed states. Multiple studies have measured blood glucose and insulin concentrations following CHO mouth rinse protocols and reported no detectable changes despite concurrent improvements in performance [92,114]. Notably, in fed-state trials where participants had consumed CHO-rich meals 2–4 hours before testing, CHO mouth rinsing did not induce any further increases in blood glucose beyond those already elicited by the meal [69,72]. An exception was reported by Ataide-Silva et al. (2016) [14], who found that although mean glucose values did not differ between CHO and placebo rinses, dynamic responses indicated that CHO rinsing helped maintain slightly higher blood glucose during prolonged exercise.

Taken together, current evidence strongly supports oropharyngeal receptor activation, rather than mucosal uptake, as the primary mechanism underlying the ergogenic effects of CHO mouth rinsing. This central neural mechanism appears to operate consistently in both fasted and fed states, reinforcing the view that its action is largely independent of circulating substrate availability. Nevertheless, our meta-analysis indicated that performance improvements were more consistent following high-CHO diets, suggesting that although the mechanism is centrally mediated, its practical expression may be more reliable when endogenous CHO stores are sufficient.

### 4.4. Strength and limitations

This systematic review and meta-analysis offered several methodological strengths that distinguish it from prior work [94–96]. First, it specifically focuses on the fed-state condition, which is an ecologically valid but underexplored area, as most existing CHO mouth rinsing studies emphasize fasted protocols. Second, it rigorously addressed statistical complexities in crossover trials by applying appropriate corrections for correlation coefficients and conducting sensitivity analyses using various *r* values. Third, data dependencies were minimized by avoiding redundant inclusion of multiple effect sizes from the same studies, thus reducing unit-of-analysis bias. Additionally, moderator and three-level subgroup analyses were used to explore heterogeneity, and Hedges’ g allowed for standardization across diverse outcomes.

Nonetheless, some limitations must be acknowledged. Although we examined pre-exercise dietary content and timing, actual CHO intake could not be standardized due to inconsistent reporting, limiting the interpretability of dietary subgroup classifications. Considerable heterogeneity was observed, and while explored through subgroup and moderator analyses, no definitive sources were identified. This may be attributed to variations in exercise protocols, participant characteristics, or imprecise correlation estimates. Egger’s test also indicated potential publication bias, which may influence effect estimates.

Furthermore, due to limited eligible studies, meta-analyses were not performed for cognitive outcomes, and direct comparisons between fed and fasted conditions could not be statistically evaluated. These areas were discussed qualitatively and warrant further targeted research. Future studies should compare nutritional states and assess CHO mouth rinsing effects on cognitive domains to improve generalizability and application.

### 4.5. Practical applications

This review provides actionable insights for athletes, coaches, and sports nutrition practitioners. CHO mouth rinsing can serve as a noninvasive, rapid ergogenic aid even in the post-meal state, with the most consistent benefits observed in aerobic exercise. Current evidence supports practical parameters such as maltodextrin-based solutions, rinse durations of 10 seconds or less, and use the following high-CHO meals, which together may maximize performance gains.

Caution is warranted when applying CHO mouth rinsing under fed conditions for anaerobic tasks, female or untrained populations, or cognitive performance contexts, where the evidence base remains limited or inconsistent. While rinse concentration showed no clear dose – response relationship, further studies are needed to establish optimal formulations tailored to specific performance goals. In elite sport, where marginal gains are highly valued, incorporating individualized trials under sport-specific conditions is recommended to determine whether CHO mouth rinsing provides a measurable competitive advantage under fed condition.

## 5. Conclusion

Despite conflicting findings, this review suggests that CHO mouth rinsing may confer small but meaningful benefits for both exercise and cognitive performance under fed conditions, although the overall certainty of evidence remains low. For exercise performance, factors such as exercise modality, rinse composition, rinse duration, and pre-exercise meal content may mediate its ergogenic effects. Specifically, CHO mouth rinsing appears to be more effective under fed conditions when applied during aerobic exercise, using maltodextrin-based solutions, rinsing for ≤10 seconds, and following a high-CHO pre-exercise meal. In contrast, evidence for cognitive outcomes is based on very few studies, highlighting the need for further research before firm conclusions can be drawn.

## Supplementary Material

Supplemental Material

## Data Availability

All data analyzed in this study were obtained from previously published studies, which are cited in the manuscript. No new data were generated for this study.
